# Engineering Antioxidants with Pharmacological Applications: Biotechnological Perspectives

**DOI:** 10.3390/antiox14091110

**Published:** 2025-09-12

**Authors:** Mădălina Paraschiv, Delia Turcov, Anca Zbranca-Toporaş, Bianca-Iulia Ciubotaru, Irina Grădinaru, Anca-Irina Galaction

**Affiliations:** 1 Department of Biomedical Sciences, Faculty of Medical Bioengineering, “Grigore T. Popa” University of Medicine and Pharmacy Iasi, 11-13 Kogalniceanu Str., 700454 Iasi, Romania; madalina.postaru@umfiasi.ro (M.P.); anca.zbranca@umfiasi.ro (A.Z.-T.); bianca-iulia.ciubotaru@umfiasi.ro (B.-I.C.); anca.galaction@umfiasi.ro (A.-I.G.); 2 Department of Inorganic Polymers, “Petru Poni” Institute of Macromolecular Chemistry, 41A Grigore Ghica Voda Alley, 700487 Iasi, Romania; 3 Department of Implantology, Removable Dentures, Technology, Faculty of Dental Medicine, “Grigore T. Popa” University of Medicine and Pharmacy Iasi, 16 University Street, 700115 Iasi, Romania; irina.gradinaru@umfiasi.ro

**Keywords:** antioxidants, oxidative stress, human health, medical biotechnology, medical bioengineering, sustainability

## Abstract

Oxidative stress, a state resulting from an imbalance between the generation of reactive oxygen species (ROS) and the body’s antioxidant capacity, is a significant contributor to the development of various human pathologies, including malignancies, cardiovascular conditions, neurodegenerative disorders, and the aging process. Antioxidants, both enzymatic and non-enzymatic, are vital in neutralizing free radicals and protecting against cellular damage. Given the limitations of synthetic antioxidants, such as potential toxicity and variable effectiveness, there has been a growing focus on biotechnological methods for producing these essential compounds. This review, titled “Engineering Antioxidants with Pharmacological Applications: Biotechnological Perspectives”, explores the latest developments in this field by examining how biological systems are being utilized to create a wide range of antioxidants. We discuss key production strategies, including the use of microbial cell factories, enzyme-driven synthesis, plant cell cultures, and metabolic engineering. The review provides specific examples of biotechnologically derived antioxidants, such as enzymatic defenses like superoxide dismutase, catalase, and glutathione peroxidase, as well as non-enzymatic molecules like carotenoids, polyphenols, and vitamins. We also evaluate the therapeutic potential of these bio-engineered antioxidants, analyzing preclinical and clinical data on their effectiveness in disease prevention and treatment. The mechanisms by which these compounds combat oxidative stress are also discussed. Finally, we address the current hurdles in scaling up production and managing costs while also outlining future research avenues, such as the creation of new production systems, advanced delivery technologies, and the discovery of novel antioxidant compounds through bioprospecting and synthetic biology. This comprehensive review highlights the potential of biotechnology to offer sustainable and impactful solutions for managing oxidative stress and enhancing overall health.

## 1. Introduction

Antioxidants are molecules that inhibit the oxidation of other substances, even when present in small amounts [1,2]. They play a vital defensive role by neutralizing the damaging oxidative processes in animal tissues and safeguarding the body from oxidative stress—a condition defined by an imbalance between the production of oxidants and the body’s ability to counteract them [2]. This overview will explore the various classifications of antioxidants, their functional mechanisms, and their widespread applications in medicine and industry.

The primary purpose of this review is to synthesize and critically evaluate the vast and often fragmented body of research concerning the production of antioxidants. While numerous studies have focused on specific aspects—such as a single extraction method, a particular microorganism, or a specific engineering strategy—a comprehensive, integrated overview that connects these disparate fields is currently lacking. This review aims to fill that gap by providing a single, cohesive resource that systematically covers the entire production pipeline, from the fundamental mechanisms of antioxidants to the most advanced biotechnological and green engineering approaches. By organizing this information thematically, this work seeks to create a clear and logical framework for understanding the current state and future direction of antioxidant science and technology.

This review is highly relevant due to the convergence of three major factors: the booming market demand for antioxidants in health and food industries, the critical scientific and industrial shift towards sustainability, and the rapid technological advancements in biotechnology and metabolic engineering. A comprehensive summary of these interconnected trends is essential for understanding the current state and future direction of the field.

This review serves as a practical and valuable resource for a diverse audience. For researchers, it consolidates current knowledge and identifies critical research gaps. For students, it acts as a structured educational tool for a complex subject. For industry professionals, it offers key insights into the latest production technologies and market trends, informing strategic decisions in process development and the adoption of more sustainable and economically viable practices.

### 1.1. Classifications of Antioxidant Compounds

The extensive family of antioxidant compounds can be organized into various categories based on their origin, functional mechanism, and physical properties like solubility.

The two major groups of antioxidants are **endogenous** antioxidants, which are synthesized by the body, and **exogenous** antioxidants, which must be acquired from external sources. The endogenous category, exemplified by enzymes like superoxide dismutase and glutathione peroxidase, represents the body’s primary line of defense against free radicals [3]. When the production of endogenous antioxidants decreases due to age, stress, toxins, and certain conditions, it is necessary to replenish the body’s reserves, reduce oxidative stress, support liver detoxification, and improve immune function.

**Figure 1:**
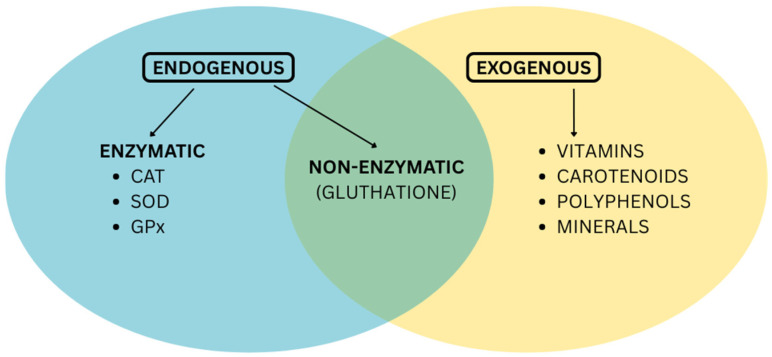
Endogenous and exogenous antioxidants.

Exogenous antioxidants, including vitamins C and E, carotenoids, and polyphenols, are obtained primarily through diet. Both categories are crucial and operate synergistically to maintain cellular balance and protect the body from oxidative stress, as seen in Figure 1.

**Figure 2:**
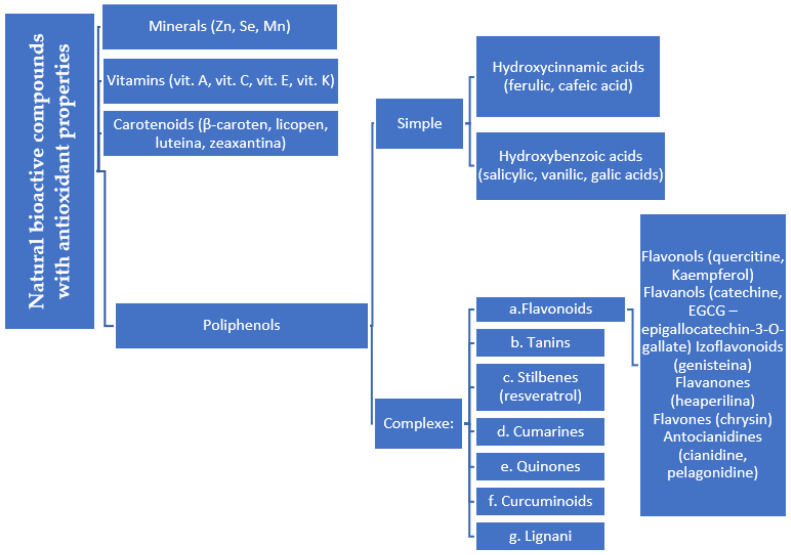
Natural antioxidants.

**Figure 3:**
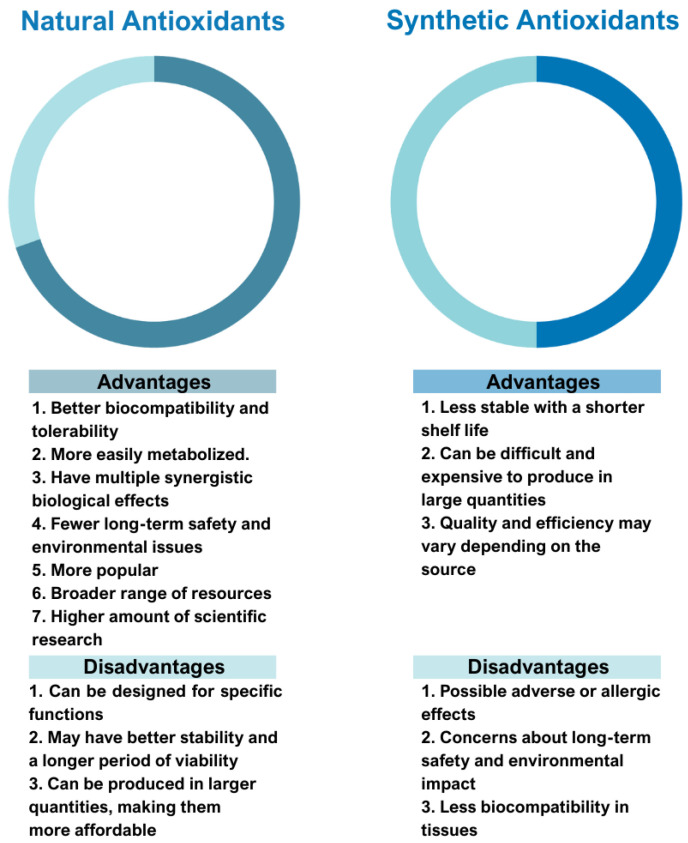
Comparative analysis of natural and synthetic antioxidants’ key features.

- Natural vs. synthetic origin—A fundamental distinction is made between antioxidants that are naturally occurring and those that are created synthetically (idebenone kinetin) [2,4,5,6]. The natural class is remarkably diverse, containing substances like vitamins, polyphenols, and terpenoids [4]. A comprehensive exemplification for natural categories of antioxidants is shown in Figure 2 and Figure 3, presenting a comparative analysis between the two classes of antioxidants.

- Enzymatic vs. non-enzymatic systems—Another key grouping separates antioxidants into enzymatic and non-enzymatic types [2,5,7,8,9].
  ∘ Enzymatic antioxidants are proteins that transform hazardous oxidative materials into safer molecules, such as water [5]. Their activity frequently relies on mineral cofactors like copper (Cu), zinc (Zn), manganese (Mn), selenium (Se), and iron (Fe) [5]. Prominent examples are superoxide dismutase (SOD), catalase, and glutathione peroxidase (GPx) [8,9]. Certain organoselenium compounds are notable for their ability to replicate the function of GPx in neutralizing reactive oxygen species (ROS) [2].
  Efficient exogenous antioxidant enzymes like SOD, catalase, or glutathione peroxidase have not been developed yet. The reason is that these are complex proteins with enzymatic functions and would be destroyed by gastric juices, becoming ineffective [10].
  ∘ Non-enzymatic antioxidants operate by halting the chain reactions initiated by free radicals [4]. This broad group encompasses vitamins, polyphenols from plants, carotenoids, and glutathione [7,8,9].

- Primary vs. secondary action—Based on their mode of action, antioxidants can be classified as either primary or secondary [6].
  ∘ Primary antioxidants, also known as chain-breaking antioxidants, directly stop oxidation by scavenging and neutralizing ROS and Reactive Nitrogen Species (RNS) [1,5].
  ∘ Secondary antioxidants employ indirect, preventive strategies [7]. Their methods include neutralizing metal ions, preventing the formation of lipid hydroperoxides, and helping to restore primary antioxidants [6].
- Other categorizations additional classifications consider properties like solubility (distinguishing between water-soluble and lipid-soluble compounds) and molecular size [5]. Whether an antioxidant is hydrophilic or lipophilic dictates where it functions within the body [8,9]. For instance, vitamin C is a water-soluble antioxidant [6], whereas vitamin E and carotenoids are fat-soluble, which is essential for protecting cellular membranes [1,7]. An emerging class of nano-antioxidants consists of nanoparticles, such as cerium and yttrium oxides, that can replicate the function of oxidative enzymes [7].

### 1.2. Antioxidant Mechanisms of Action

Antioxidant molecules can function through various chemical pathways [6]. The specific mechanism—or combination of mechanisms—a molecule uses is dictated by its unique chemical structure [1].

- Hydrogen Atom Transfer (HAT)—In the HAT mechanism, an antioxidant neutralizes a free radical by transferring a hydrogen atom to it [1,2,8,9]. This is a dominant mechanism for polysaccharides that possess hydroxyl groups and for phenolic compounds [6]. The antioxidant radical that forms as a result is typically less reactive because its structure is stabilized by resonance [8].
- Single Electron Transfer (SET)—Through the SET mechanism, an antioxidant provides an electron to a free radical, an action that can bring oxidative chain reactions to a halt [1,2,8,9]. Carotenoids possess a strong capacity for electron donation [6], and plant-derived compounds such as phenols and flavonoids utilize this pathway to neutralize radicals and reduce metal ions [9]. The prevalence of HAT versus SET can be influenced by the solvent environment [1,2]. A related process, Sequential Proton Loss Electron Transfer (SPLET), can be the main mechanism in non-aqueous environments [7].
- Metal Chelation—Antioxidants often work by binding to transition metals in a process called chelation [5,8,9]. Ions of metals like iron (Fe) and copper (Cu) can act as catalysts in the creation of radicals [7]. By sequestering these metals, antioxidants like phenolic compounds can block the start of oxidative processes [1,7].
- Modulation of Cellular Pathways—Certain antioxidants work by influencing biological processes at the cellular level. For example, polyphenols can trigger the body’s own production of antioxidant enzymes, including catalase, SOD, and GPx [11]. They are also capable of blocking enzymes that generate free radicals, like xanthine oxidase [11]. Furthermore, tocotrienols, which are a form of vitamin E, can inhibit the inflammatory transcription factor NF-κB [12,13,14].

### 1.3. Applications and Uses of Antioxidants

Antioxidants are utilized in a wide variety of fields, including healthcare, the food sector, materials science, and the cosmetics industry [4], as shown in Table 1.

**Table 1:** Applications of antioxidants.

Application Area	Benefits
Managing Chronic Conditions	
Cardiovascular Health	• Preventing illnesses like atherosclerosis [1,5].
	• Aiding in lowering cholesterol [8].
Cancer	• Inhibiting tumor cell growth and triggering apoptosis (programmed cell death). • Blocking angio-genesis (formation of new blood vessels for tumors) [1,5,8].
Neurodegenerative Disorders	• Combating oxidative stress and neuroinflammation in conditions like Parkinson’s disease. • Aiding in preventing neurodegenerative conditions (e.g., vitamin A) [3,15].
Diabetes	• Helping manage the condition by restoring depleted antioxidant levels [2,5,8].
Dermatology and Skin Care	
Skin Health and Treatment	• Slowing skin aging and preventing the breakdown of collagen. • Shielding skin from UV damage. • Treating skin disorders like psoriasis and acne. • Aiding in collagen synthesis (e.g., vitamin C) [2,4,16].
Immune Support and Infectious Diseases	
Antimicrobial Activity	• Effective against pathogenic bacteria like E. coli and S. aureus (e.g., plant-based silver nanoparticles) [5].
Antiviral Activity	• Showing activity against various coronaviruses (e.g., flavonoids). • Helping manage symptoms of viral infections (e.g., vitamin C) [5,8].
Industrial and Therapeutic Fields	
Targeted Therapeutics	• Treating oral conditions like periodontal disease [7]. • Helping manage drug-induced liver damage and Non-Alcoholic Fatty Liver Disease (NAFLD).
Advanced Technologies	• Used in Drug Delivery Systems (e.g., polysaccharides). • Applied in nanotechnology as reducing agents for creating silver and gold nanoparticles [9].

Antioxidant compounds work by influencing key molecular pathways to prevent and treat various diseases. Their mechanisms often involve neutralizing harmful free radicals, regulating the activity of enzymes, and controlling cellular processes like proliferation and apoptosis. The precise molecular mechanisms of some of the most popular antioxidants and the underlying molecular pathways and mechanisms in relation to specific disease categories are presented in Table 2.

**Table 2:** Specific mechanism of action of antioxidant compounds.

Compound (Origin)	Type/Class	Antioxidant Mechanism of Action	Other Biological Effects
**Idebenone** (synthetic)	Analogue of ubiquinone (CoQ10)	Idebenone captures free radicals and inhibits lipid peroxidation. It is considered to transfer electrons directly to complex III of the mitochondrial electronic transfer chain, restoring cellular energy generation (ATP). Unlike CoQ10, idebenone can bypass dysfunctional complexes in the ETC, ensuring continued energy production even under conditions of oxidative stress [17].	Protects against neurodegenerative and cardiovascular diseases Antiaging, reduces skin photoaging
**Ubiquinone (Coenzyme Q10)** (All commercial sources of ubiquinone utilized in topical products are synthetically derived. For nutritional supplements, the fermentation processes of *Agrobacterium tumefaciens are used*.)	Polyphenols—quinone	In its oxidized form (ubiquinone), it acts as an electron and proton carrier in the **mitochondrial electron transport chain (ETC)**. It transfers electrons from dehydrogenases to complex III, playing a vital role in cellular energy production in the form of ATP. In its reduced form (ubiquinol), acts as a potent **antioxidant** by scavenging free radicals and protecting cellular components from oxidative damage. The reduced form also regenerates vitamin E (donates an electron to the α-TO·. This donation regenerates the active α-TOH, allowing it to continue its role as a primary antioxidant) [18].	Supports cellular regeneration, tissue restoration and elastin and collagen synthesis Reduces DNA damage from keratinocytes and the production of UVA-induced metalloproteinases in fibroblasts
**Vitamin E** (natural—oily plants (rape, sunflower, soybean, corn, oil, seeds))	Vitamin—tocopherols, tocotrienols	Vitamin E donates a hydrogen atom and becomes an unreactive tocopheroxyl radical, which can be regenerated by other antioxidants. It neutralizes singlet oxygen in the cell membrane. It prevents lipid peroxidation (oxidation of unsaturated fatty acids such as arachidonic acid in the phospholipid membrane) [13].	Cardioprotective and antitumor effects; prevents cataracts, neurodegenerative diseases, and arthritis
**Vitamin C** (natural—fruits, vegetables)	Vitamins	Vitamin C is a one-electron donor, leading to the extensive removal of free radicals. It contributes to the regeneration of oxidized vitamin E (chemical property of being able to transition between its reduced (L-ascorbic acid) and oxidized (dehydroascorbic acid or DHAA) forms). Critical cofactor for enzymes involved in collagen synthesis, such as procollagen-proline dioxygenase and procollagen-lysine dioxygenase [19].	Supports immune functions Cofactor involved in collagen synthesis Inhibits melanogenesis, induces collagen synthesis, supports the production of skin-specific lipids, and has neuroprotective effects
**Resveratrol** (natural—*Vitis vinifera* sp., *Polygonum cuspidatum*)	Polyphenols—Stilbene	Resveratrol inhibits the release of interleukin-8. It blocks the expression of COX-2 and the biosynthesis of prostaglandin D2 (PGD-2). It induces the antioxidant enzyme system and is a cell cycle regulator. It inhibits the damage and mutagenic action of DNA. At lower doses, it is an anti-apoptotic agent, providing cardioprotection by activating survival signals (upregulation of nitric oxide (NO) synthesis). At higher doses, it acts as a pro-apoptotic agent, inhibiting the synthesis of RNA, DNA, and proteins, causing chromosome aberrations, and blocking cell proliferation. It has inhibitory effects on protein kinase C (PKC) and tyrosine kinase (often activated in tumors) [20,21,22].	Anti-inflammatory and antitumor action Stimulates detoxification Antimicrobial, antiviral, and antifungal action Inhibits the proliferation of keratinocytes
**Lycopene** (natural—tomatoes)	Carotenoid	Lycopene effectively removes free radicals and has a powerful singlet-oxygen-quenching ability. It modulates various signaling pathways, including those for growth factors like insulin-like growth factor-1 (IGF-1) (a key factor in tumor development and metastasis). It also upregulates the expression of a gene called connexin 43, which improves intercellular gap junction communication, a function often deficient in tumors. Activates certain detoxification enzymes, known as Phase II enzymes, which help to neutralize carcinogens. Lycopene prevents the oxidative modification of low-density lipoproteins (LDLs) [23].	Antitumor properties; prevents atherosclerosis and ophthalmological diseases
**Lutein** (natural—vegetables)	Carotenoid—xantophylls	Lutein protects fibroblasts from UVA-induced oxidation and prevents reduction of catalase (CAT) and superoxide dismutase (SOD) enzymes. It is more stable in the action of oxidation than other carotenoids such as beta-carotene and lycopene [8,24].	Anti-inflammatory properties; protective ofeye tissue
**Ferulic acid** (natural)	Hydroxy-cinnamic acids—polyphenolic compounds	Ferulic acid directly neutralizes free radicals. It forms stable phenoxyl radicals. It inhibits enzymes that generate free radicals and enhances the activity of other antioxidant enzymes, such as superoxide dismutase (SOD) and catalase. It inhibits enzymes like cyclooxygenase-2 and xanthine oxidase and reduces ROS production, preventing the downstream signaling that leads to inflammation. It suppresses the activation of NF-κB and reduces the expression of pro-inflammatory cytokines (TNF-α, IL-6). It counteracts nicotine-induced toxicity by increasing the body’s endogenous antioxidant defenses and quenching free radicals [25,26].	Antimicrobial, anti-inflammatory, antithrombotic, and antitumor action; vascular rotector
**Pycnogenol** (extract) (natural—*Pinus pinaster* ssp. *Atlantica)*	Phenolic compounds (catechins, epicatechins and taxifolin), flavonoids (proanthocyanidins), phenolic acids (cinnamic acids and other glycosides)	Pycnogenol increases the synthesis of antioxidant enzymes and protects other antioxidants (vit. C. E, glutathione) [23].	Reduces blood pressure Increases the level of glucose in the blood Relieves asthma and symptoms of allergic rhinitis Improves lung function
**Quercetin** (natural)	Flavonoid	Quercetin regulates glutathione and its action and inactivates free radicals. It donates a hydrogen atom, neutralizes the toxic effect of singlet oxygen by inactivating its excitation energy state, and prevents lipid peroxidation [23].	Prophylactic potential in osteoporosis, some types of tumors, and lung and cardiovascular conditions
**Kaempferol** (natural)	Flavonoid	Kaempferol reduces superoxide anion, hydroxyl radical, and peroxyinitrite levels [23].	Antitumoral Anti-inflammatory Antiproliferative
**Crocin** (natural—saffron (*Crocus sativus*))	Carotenoid	Crocin reduces the level of several pro-oxidants and stimulates SOD and glutathione peroxidase activity (GPX) [23].	Anti-inflammatory Immunomodulator Neuroprotective Antidepressant
**Caffeic acid** (natural)	Phenolic compounds—hydroxy-cinnamic acids	Caffeic acid works via relocation of unpaired electrons into the extended conjugated side chain [23].	Anti-inflammatory, antitumor, antibacterial, and antifungal action; prevents neurodegenerative diseases; prevents toxicity in chemotherapy
**Caffeine** (Natural)	Methylxanthin alkaloid	Caffeine is a small-molecule activator of sirtuin 3 (SIRT3), a major mitochondrial deacetylase. This enhances its enzymatic activity, which in turn leads to the deacetylation and activation of superoxide dismutase (SOD). It activates the peroxisome proliferator-activated receptor (PPAR) pathway, contributing to repairing damage from oxidative stress [27,28].	Stimulator of the central nervous system Improves muscle contractility
**Niacinamide** (vitamin B3)—nicotinic acid and nicotinamide (predominantly synthetic)	Water-soluble vitamin	Niacinamide is a precursor for the essential coenzyme NAD+, which is critical for redox reactions and energy production in cells. It decreases the activity of enzymes that produce free radicals, such as NADPH oxidase and nitric oxide synthase (NOS). It also increases the activity of antioxidant enzymes like catalase and superoxide dismutase, which neutralize harmful molecules like hydrogen peroxide and superoxide radicals. It improves the accumulation of intracellular calcium ions [29].	Antioxidant—protects keratinocytes from oxidative stress

The structure of a molecule dictates its function, and in the case of antioxidants, specific recurring features are responsible for their ability to neutralize harmful free radicals in the ways presented above. Table 3 summarizes the chemical structures of the most common types and groups of antioxidants in 2D images.

The key ideas related to the chemical structures of these common antioxidants can be summarized as follows:

✓ **Phenolic rings and hydroxyl groups have the central role.** They are aromatic rings with at least one attached hydroxyl (–OH) group. This feature is the foundation for entire classes of antioxidants like **flavonoids (quercetin**, **kaempferol**, **genisteine**, and **epigallocatechin gallate (EGCG))** and **phenolic acids (ferulic acid** and **caffeic acid)**.

**As for other phenols**, **resveratrol** is a stilbenoid composed of two phenol rings, and **vitamin E** has a crucial hydroxyl group on its chromanol ring that is its active site.

The hydroxyl group is critical because it can easily donate a hydrogen atom to a free radical, neutralizing it. The resulting antioxidant radical is stabilized by the delocalization of the unpaired electron around the aromatic ring, making it relatively non-reactive.

✓ **The importance of conjugated double bonds is related to** the presence of an extensive **conjugated system**, which consists of alternating single and double bonds. This is most prominent in the **carotenoid** family (**lycopene**—highly effective at quenching singlet oxygen, a particularly reactive type of free radical; **lutein and zeaxanthin** similar to lycopene but classified as xanthophylls because they have hydroxyl groups on their terminal rings).
✓ **The impact of structure on solubility** dictates where the compound acts in the body. **Lipophilic (fat-soluble)** molecules with long hydrocarbon chains, like **coenzyme Q10**, **vitamin E**, **lycopene,** and **lutein**, can operate within the lipid environment of cell membranes.

**Hydrophilic (water-soluble)** compounds with numerous polar functional groups, such as the many hydroxyl groups in **vitamin C** and the sugar units in **crocin**, are soluble in aqueous environments like blood and the cytoplasm.

- Managing chronic and degenerative conditions—Oxidative stress is a contributing factor in many diseases, such as cancer, arteriosclerosis, and diabetes [1]:
  ∘ Cardiovascular health—The potential of antioxidants to prevent illnesses such as atherosclerosis [2,12] and myocardial infarction [1] is a significant area of study. Vitamin E tocotrienols aid in this by obstructing HMG-CoA reductase, an important enzyme for cholesterol production [30]. Similarly, a diet rich in carotenoids is associated with a reduced risk of cardiovascular problems [5].
  ∘ Cancer—Research indicates that tocotrienols can inhibit the growth and trigger the programmed death (apoptosis) of various cancer cells, including breast, colon, and lung tumors. These compounds also inhibit angiogenesis, the process by which tumors create new blood vessels to sustain themselves [30].
  ∘ Neurodegenerative disorders—Oxidative damage is a key element in conditions such as Parkinson’s and Alzheimer’s disease [5,31]. Consequently, polyphenols like resveratrol, curcumin, and quercetin are being explored as potential therapeutic alternatives for Parkinson’s [11], as they can address both oxidative stress and neuroinflammation [11]. Vitamin A is also recognized for its role in nutritional strategies aimed at preventing neurodegenerative conditions [5].
  ∘ Diabetes—Antioxidants are investigated for their utility in the management of diabetes [2,5,31]. For instance, polysaccharides from the G. lucidum mushroom were observed to help restore diminished levels of non-enzymatic antioxidants in diabetic rat models.
- Dermatology and skin care—Antioxidants are common ingredients in cosmetic products designed to slow skin aging [4] and treat skin disorders associated with oxidative stress, such as photoaging, psoriasis, and acne [8]. They help shield the skin by forming a barrier against UV damage and by preventing the breakdown of collagen [4]. Vitamin C plays a vital role by aiding in collagen production and helping to restore the antioxidant function of vitamin E after it has been oxidized [8].
- Immune support:
  ∘ Antimicrobial activity—Potent antimicrobial effects have been observed in bioactive compounds derived from fermented koji rice [15]. Additionally, silver nanoparticles created with plant extracts have proven effective against pathogenic bacteria such as *E. coli* and *S. aureus* [9].
  ∘ Antiviral activity—Powerful antiviral capabilities have been identified in flavonoids like quercetin and catechin, including activity against several types of coronaviruses [6]. The use of vitamin C supplements increased notably during the COVID-19 pandemic due to their perceived ability to help manage symptoms of viral infections [12].
- Other industrial and therapeutic fields—Antioxidants are applied in treating oral conditions like periodontal disease [5], as well as liver damage induced by drugs and nonalcoholic fatty liver disease (NAFLD) [13]. Polysaccharides with antioxidant functions have demonstrated potential for anticancer, anti-inflammatory, and blood sugar-lowering effects [14] and are incorporated into drug delivery technologies [14]. In the field of nanotechnology, antioxidants sourced from plant extracts serve as reducing agents for creating silver and gold nanoparticles [9,32,33].

### 1.4. Trends and Challenges

While antioxidants are recognized for their benefits, the process of bringing them from their origin to the market is filled with considerable difficulties. In parallel, the global antioxidant sector is undergoing significant expansion and a notable pivot toward sustainable practices, propelled by consumer interest and technological progress. This chapter aims to outline the main obstacles in the manufacturing of antioxidants and to examine the dominant economic and technological developments that are defining their future.

#### 1.4.1. Obstacles in the Supply and Manufacturing of Antioxidants

The mass production of antioxidants is hindered by multiple issues spanning sourcing, extraction methods, chemical synthesis, and biological effectiveness.

##### Difficulties in Sourcing and Extraction

Procuring antioxidants from natural origins is accompanied by several challenges:

- The collection of plant-based materials is frequently limited by issues like the endangerment of species from over-harvesting, slow maturation rates, and availability that changes with the seasons [5,6,31].
- The consistency, output, and effectiveness of the final antioxidant product can fluctuate greatly depending on the environment and the specific techniques used for farming, collecting, and processing [8].
- The concentration of the desired compounds is often exceptionally low; for instance, secondary metabolites typically represent less than 1% of a plant’s total dry weight [5,6,31].
- Established extraction methods face criticism for being expensive, inefficient, and requiring significant time [15]. These processes demand substantial energy and solvent use and risk degrading compounds that are sensitive to heat [5,6,31,34].
- The enzymatic action of polyphenol oxidase (PPO) during extraction can also lead to browning, which degrades the valuable polyphenols [5,6,31].

##### Issues with Bioavailability and In Vivo Performance

A critical hurdle is the discrepancy between how an antioxidant behaves in a lab setting and how it performs inside a living system [2,5,6,31]:

- A high level of reactivity observed during in vitro experiments does not guarantee effectiveness in vivo [2,5,6,31].
- A large number of antioxidants, such as polyphenols and tocotrienols, exhibit limited bioavailability. This is often due to inefficient absorption, poor solubility, or breakdown within the stomach [5,6,7,8,9,12,13,14,15,31,33].
- Because free radicals have an exceptionally brief existence, it is challenging for an antioxidant to be available at the exact moment and location where oxidative damage occurs [1].
- There is a recognized necessity for thorough clinical trials to confirm the safety and efficacy of antioxidants and to establish the optimal dosages for treating specific illnesses, like the various forms of Parkinson’s disease [11].
- Taking high doses in supplements may even pose health risks, as suggested by some scientific studies [5,6,31].

##### Obstacles in Synthesis and Production

Both chemical and biotechnological manufacturing pathways present their own problems:

- Chemical synthesis can be costly and frequently results in unwanted secondary products [5,6,31].
- Established chemical synthesis routes often depend on the use of toxic reducing and stabilizing chemicals, which restricts the use of the finished product in medical and biological applications [8,9,30,33].
- For vitamin C, the long-standing Reichstein process is known for its high energy consumption, reliance on dangerous operating conditions, and difficulties with waste management [12,13,14].
- While more environmentally friendly biotechnological options are emerging, they are not without their own challenges. For instance, no known natural microbe can complete all the required steps for vitamin C production in a single fermentation [12,13,14]. Boosting the output of these microbial systems continues to be a difficult task [12,13,14].
- Within agriculture, enhancing the vitamin E levels in crops via breeding is a key objective, but it is complicated by the complexity of the underlying genetic mechanisms [12,13,14].

##### Complexities in Structure and Methodology

The sophisticated nature of certain antioxidants poses distinct hurdles for research and development:

- In the case of polysaccharides, their intricate structures, along with the constraints of available analytical techniques, have meant that research in this area has not kept pace with that of proteins and nucleic acids [14]. It is still challenging to clearly define the relationship between the structure of a polysaccharide and its ability to act as an antioxidant [14].
- Moreover, because various antioxidants operate through different mechanisms, direct comparisons of activity results from one study to another is difficult [8,9,33].

#### 1.4.2. Market Developments and Economic Outlook for Antioxidants

Although these production challenges, the antioxidant industry is growing rapidly, influenced by evolving consumer tastes, technological breakthroughs, and an increasing focus on sustainability.

#### Increased Demand and Market Growth

The economic trajectory for antioxidants is ascending:

- Antioxidants have achieved widespread recognition through media exposure [14], while scientific investigation into their health-promoting capabilities has surged since the 1990s [5].
- A major driver of this growth is a strong consumer shift toward natural ingredients instead of synthetic alternatives, motivated by concerns about personal health and the environment [5,6,11,31].
- The worldwide market for plant-based extracts, which is heavily influenced by the cosmetics sector, was estimated at USD 10.19 billion in 2021, with projections expecting it to climb to USD 22.49 billion by 2030 [5,6,31].
- Certain antioxidant-based items, like Lentinan and polysaccharides extracted from the *Ganoderma lucidum mushroom* (“Ling-Zhi”), have already been brought to market as successful pharmaceutical agents [14].

#### A Move Toward Green Technology and Sustainable Practices

A clear trend is the industry’s adoption of sustainable and environmentally conscious methods [8]:

- There is a rising inclination toward using “green synthesis” techniques, which are favored for being eco-friendly and for avoiding the use of hazardous chemicals [8,9,33].
- Advanced green extraction methods, one example being microwave-assisted extraction (MAE), are being adopted because they are more cost-effective, faster, and more sustainable compared to older techniques [15]. Such green technologies have been the subject of over 200 patent filings globally [15].
- In the biotech sector, a prominent development involves using low-cost raw materials, such as waste from agriculture, as a food source for microbes in fermentation processes to create vitamins, thereby making production more economical and greener [12].

#### Sustainability and Upcycling of By-Products

The concept of a “zero-waste” or circular economy is being progressively integrated into the production of antioxidants [5,6,11,31]:

- A notable development is the practice of extracting valuable antioxidants from the waste materials of food production, including fruit peels, seeds, and pomace [5,6,31].
- This strategy serves a dual purpose: it mitigates the negative economic and environmental effects of waste while simultaneously generating new, valuable products [5,6,31].
- These recovered polyphenols are finding new life as natural preservatives in foods to slow down lipid oxidation, as agents to prevent browning, as natural colorants, and as active components in smart food packaging that indicates freshness [5,6,31].

### 1.5. Advances in Research and New Product Directions

The focus of scientific research is also shifting.

**Specific research advancements as solutions to sourcing, extraction and formulation difficulties** for more controlled and sustainable methods are outlined below.

#### 1.5.1. Biotechnological Synthesis

- **Strain engineering**: Using protein engineering and copy number engineering to enhance L-AA production in yeast, researchers have successfully **reconstructed a vitamin C biosynthesis pathway** in *Saccharomyces cerevisiae*. A key finding from a study on *S. cerevisiae* was **the identification of rate-limiting steps in the vitamin C pathway.** By fusing enzymes like L-GalDH and L-GLDH or overexpressing the rate-limiting enzyme GPP, they achieved an increase in L-AA production and accumulation [19].

The “one-step fermentation” method produces vitamin C directly from glucose without the need for the 2-keto-L-gulonic acid (2KGA) intermediate used in older methods:

- **Substrate innovation**: A promising new direction involves utilizing agricultural waste as a substrate for fermentation. For example, a novel strain of *Gluconobacter oxydans* was isolated and identified as a potent L-AA producer that could be adapted to grow on hydrolysates from plant waste like mango leaves. The L-AA yield from this method was further enhanced by treating the bacterial strain and the waste material with low doses of gamma radiation, boosting the yield [14].

#### 1.5.2. Innovative Sourcing and Extraction

- **Green extraction techniques**—parallel to fermentation, there has been a push for “green extraction” methods from natural sources like fruits, vegetables, and algae, without large amounts of harmful solvents. These include **ultrasound-assisted extraction (UAE)**, **microwave-assisted extraction (MAE)**, **pressurized liquid extraction (PLE)**, and **supercritical fluid extraction (SFE)**. These methods are more environmentally friendly, consume less energy, use mild conditions, and can reduce processing times while achieving high extraction yields. For example, SFE uses carbon dioxide as a solvent, which is non-toxic and easily removed from the final product.

This approach also allows for the **valorization of waste products**, such as orange and pomegranate peels, which are rich in L-AA [35]. A key trend is the use of agricultural and food industry by-products, often referred to as “waste”, as a source for antioxidants. This circular economy approach not only reduces environmental waste but also provides a cheap and abundant source of valuable compounds. For example, methods have been developed to extract high-purity lycopene from tomato processing waste, such as peels and seeds, for use in food and nutraceuticals. This has been applied to other antioxidant-rich by-products like fruit peels, seeds, and pulp.

The actual obstacles and challenges in the production field (sourcing and extraction) are

- **Scaling up biotechnological processes**: While one-step fermentation of antioxidants like vitamin C in engineered strains like *Saccharomyces cerevisiae* is a promising advance, it is not yet industrially scalable due to stability challenges.
- **Controlling yield and purity**: Extracting natural antioxidants from plants, even with modern techniques, is difficult and often suffers from low yields and the presence of impurities. For example, the yield of pure resveratrol from the root of *Polygonum cuspidatum* is so low that 1.5 kg of roots gives approximately 1 mg of resveratrol. Similarly, some green extraction methods, like ultrasound-assisted extraction of lycopene, do not always outperform conventional methods in terms of yield and activity.
- **Minimizing degradation**: Many natural antioxidants, such as lycopene and ferulic acid, are sensitive to light, heat, and oxygen, making them prone to degradation during extraction and processing. This presents a significant challenge to maximizing the nutritional value of final products.

**Future direction for process scaling up to an industrial level:** A significant trend in agriculture is the biofortification of major food crops, which aims to boost their nutritional profile, such as by increasing their vitamin E content through both traditional breeding and modern transgenic methods [31].

Further studies are needed to address challenges in stability and to create robust genetic circuits that can perform consistently in complex, real-world industrial environments.

**Future direction for minimizing degradation:** Future research should focus on understanding the chemical stability mechanisms of antioxidants like lycopene and on designing low-cost, high-efficiency production and encapsulation systems that protect the compounds from degradation.

#### 1.5.3. Nanoparticle-Assisted Synthesis

The use of nanotechnology is emerging as a way to produce antioxidants and enhance their properties.

- **Green nanoparticle synthesis**: A study successfully biosynthesized silver nanoparticles (AgNPs) using an aqueous leaf extract from the medicinal plant *Decaschistia crotonifolia*. The plant’s bioactive compounds, such as flavonoids and polyphenols, acted as both reducing and capping agents for the nanoparticles. The resulting AgNPs showed excellent antioxidant activity against DPPH, hydrogen peroxide, and nitric oxide radicals, with higher scavenging activity than the raw plant extract alone.

#### 1.5.4. Improved Product Formulation and Delivery

Unfulfilled expectations from some clinical trials involving tocopherols have prompted an increased scientific curiosity in tocotrienols [30], which have been dubbed the “vitamin E of the 21st century” because of their distinct molecular interactions and promising health advantages [30].

There is a growing push to create multifunctional antioxidants capable of effectively regulating and preserving the body’s oxidative balance [13]

To overcome challenges with stability and bioavailability, new delivery systems are being developed.

- **Nanotechnology-based delivery**—A review on antioxidants highlights the use of nanotechnology-based delivery systems to improve the bioavailability of active compounds: nanoparticles, liposomes, and polymeric micelles can protect active molecules from degradation, improve their solubility, and enable targeted delivery to specific tissues.
- **Derivatives and synergistic combinations**—For example, ferulic acid is often used with vitamins C and E to create topical solutions that offer increased photoprotection for the skin. The combined effects can provide benefits that a single compound cannot achieve alone. The development of resveratrol analogs, like resveratryl triacetate (RTA) and resveratryl triglycolate (RTG), has been shown to improve stability and efficacy in cosmetic formulations. Additionally, a study found that combining resveratrol with y-tocotrienol acted synergistically to provide a greater degree of cardioprotection than either compound alone. This effect was linked to the activation of the Akt-Bcl-2 survival pathway and the induction of autophagy.
- **Targeted delivery**—New formulations are being designed for specific applications, such as tumor-targeted drug delivery, using antioxidant nanoparticle polymers.

Considering medical bioengineering and pharmacology, **physiological and clinical understanding represent distinct, consistent directions of research on the dosage, unexpected side effects (including pro-inflammatory ones), low bioavailability, lack of specificity in delivery, variable efficacy of delivery systems, and complex interactions with other food supplements of antioxidants.**

## 2. Materials and Methods

This review synthesizes findings from a comprehensive survey of scientific research concerning the production of antioxidants. The methodology was designed to identify, select, and organize relevant primary research articles, review papers, and patents.

A systematic literature search was conducted using major scientific databases.

Information was extracted from the final pool of selected articles and organized thematically. The collected data was synthesized and structured into the main chapters of this review, covering the fundamental categories and applications of antioxidants, the primary production paradigms (natural extraction, chemical synthesis, microbial and plant-based biotechnology), the specific microorganisms and engineering disciplines involved, and the overarching challenges and future trends in the field.

The vast majority of the references are concentrated in the last decade, particularly from 2017 to 2024, as the review is heavily focused on the latest advancements and current state of research in the field of antioxidants.

## 3. Microbial Fermentation as a Platform for Antioxidant Production

### 3.1. An Overview of Microbial Bioproduction

The use of microorganisms for producing high-value antioxidants stands as a leading biotechnological field [36,37]. This approach offers a more sustainable and economically viable pathway compared to traditional chemical synthesis methods [38,39,40,41,42,43]. A wide array of microbes, including bacteria, fungi, yeasts, and microalgae, serve as effective “cell factories” [5,44]. Their main advantages lie in their rapid growth rates within highly controlled and scalable bioreactor systems, positioning them as an ideal source for the industrial manufacturing of antioxidants destined for pharmaceutical and food applications [5,45].

### 3.2. Manufacturing of Key Antioxidants Using Microbial Systems

#### 3.2.1. Vitamin C (Ascorbic Acid)

Microbial fermentation is central to the industrial manufacturing of vitamin C and its immediate precursors:

- The Two-Step Fermentation Process: This is the prevailing industrial methodology for synthesizing 2-keto-L-gulonic acid (2-KLG), the direct precursor to vitamin C [35,36,37]. It consists of two distinct microbial transformations:
  1. First, the bacterium Gluconobacter oxydans performs a highly efficient bioconversion of D-sorbitol into L-sorbose [24,36].
  2. Next, a mixed-culture fermentation converts the L-sorbose into 2-KLG [24,36]. This requires a symbiotic relationship between a producing strain, Ketogulonicigenium vulgare, and a “companion strain” such as Bacillus megaterium [37,46]. The companion microbe is vital as it provides essential metabolites that promote the growth of K. vulgare, thus boosting the overall production of 2-KLG [24,36,37].
- The One-Step Fermentation Goal: A more streamlined objective is to produce vitamin C directly from a simple carbohydrate like D-glucose within a single fermentation process [36,47]. Current research is aimed at achieving this by genetically modifying yeasts like Saccharomyces cerevisiae to contain the complete biosynthetic pathway [35,48].

#### 3.2.2. Glutathione

The principal biotechnological route for glutathione production is microbial fermentation [49,50,51].

- Key Microbial Producers:
  ∘ Saccharomyces cerevisiae, commonly known as baker’s yeast, is the leading commercial microorganism for industrial glutathione manufacturing [49,50,51]. Its GRAS (Generally Recognized as Safe) designation makes it particularly suitable for products in the food and medical sectors [49,50,51].
  ∘ Other yeast species, including Candida utilis and Pichia pastoris, are also recognized as potent glutathione producers [49,50,51].
  ∘ Through genetic engineering, Escherichia coli has been transformed into a highly productive host for manufacturing glutathione [49,50,51].
- Manufacturing Process: Glutathione is synthesized and stored inside the microbial cells (intracellularly) during the fermentation run [49,50,51].To maximize output, a fed-batch cultivation strategy is commonly implemented, where nutrients are supplied incrementally to support high cell densities [49,50,51].The yield can be further increased by supplementing the culture medium with the three precursor amino acids required for glutathione synthesis [38,39,40,41,42,43].

#### 3.2.3. Carotenoids

A broad spectrum of microorganisms naturally synthesize carotenoid antioxidants [34].

- Natural Microbial Sources:
  ∘ Yeasts: Species such as *Phaffia rhodozyma* (also called *Xanthophyllomyces dendrorhous*) and Rhodotorula are well-known for producing β-carotene and astaxanthin [44,52].
  ∘ Fungi: The genera Blakeslea and Mucor are notable for their production of lycopene and β-carotene [34].
  ∘ Bacteria: Paracoccus and Gordonia are among the bacterial genera that can synthesize carotenoids [44].
  ∘ Microalgae: This group is a primary source of carotenoids, including *Haematococcus pluvialis* for astaxanthin and *Dunaliella salina* for β-carotene [44,53].
- Manufacturing Process: The production of microbial carotenoids occurs in bioreactors where conditions like pH, temperature, and nutrient levels can be precisely managed to optimize the yield [34]. The type of carbon source used in the fermentation medium is a decisive factor that influences both production costs and final output [34].

#### 3.2.4. Coenzyme Q10 (Ubiquinone)

Microbial fermentation is the preferred biotechnological pathway for CoQ10 production, offering advantages over chemical synthesis [16].

- Key Microbial Producers: Many bacteria and yeasts can synthesize CoQ10 [32]. The most prominent bacterial producers are found in genera like Agrobacterium, Rhodobacter, and Paracoccus [18,54]. Strains of Agrobacterium tumefaciens and Rhodobacter sphaeroides have been particularly optimized for high-yield industrial manufacturing [18,54,55,56].
- Manufacturing Process: CoQ10 is typically produced using a high-density fed-batch fermentation strategy [54,55,57]. This approach allows for the controlled addition of nutrients throughout the process to foster dense cell growth and high product yields [55,57]. The optimization of culture parameters is essential, with the maintenance of high dissolved oxygen levels being particularly critical, as the biosynthesis of CoQ10 is an aerobic process [54,55,56].

#### 3.2.5. Vitamin E Precursors and Other Lipophilic Antioxidants

While industrial fermentation does not produce vitamin E directly, it is used to create essential precursors [36,58].

- A state-of-the-art process involves using engineered Saccharomyces cerevisiae to ferment glucose into β-farnesene [33]. This microbially derived farnesene is subsequently used as a starting material for the chemical synthesis of isophytol, a primary component of synthetic vitamin E [58,59].
- Genetically modified yeasts are also harnessed to produce other lipophilic antioxidants, including stilbenoids like resveratrol and various carotenoids [36]. Different cultivation strategies are employed to enhance productivity:
  ∘ Batch Fermentation: This simpler method has been used to generate products like lycopene [36].
  ∘ Fed-Batch Fermentation: This technique has enabled high-titer production of resveratrol and β-carotene in the yeast Y. lipolytica [36].
  ∘ Multistage Fermentation: This advanced strategy decouples the cell growth phase from the product synthesis phase to improve efficiency. For example, two-stage and three-stage fermentation systems have been successfully designed for producing astaxanthin, tocotrienols, and lutein [36].

#### 3.2.6. Nicotinamide and Nicotinic Acid (Vitamin B3 Forms)

Microbial biotransformation, particularly using whole-cell biocatalysts, is a significant route for manufacturing nicotinamide and nicotinic acid [60,61].

- Key microbial producers—Bacteria from the Rhodococcus (e.g., Rhodococcus rhodochrous), Nocardia, and Pseudomonas genera are known for their enzymatic ability to perform the necessary conversions [60,61].
- Process overview—The production involves growing the selected microorganisms in a controlled liquid fermentation medium. The harvested microbial cells, which are rich in the required enzymes, are then used as whole-cell catalysts to convert the substrate 3-cyanopyridine into the final nicotinamide product [37].

### 3.3. Overarching Strategies in Microbial Antioxidant Production

#### 3.3.1. Upcycling of Food and Agricultural By-Products

A key green strategy in modern biotechnology is using microbial fermentation to add value to low-cost agro-industrial by-products [44,62,63].

- Solid-state fermentation (SSF): This sustainable technique involves cultivating microorganisms on solid substrates like fruit pomace with minimal free water [62,63]. The microbes secrete enzymes that break down plant cell wall structures, which in turn releases bound phenolic compounds and enhances the material’s overall antioxidant activity [62,63].
- Submerged fermentation (SmF): This method cultivates microbes in a liquid medium enriched with the food by-product, which serves as a nutrient source [62,64].
- Microbes like the fungus *Aspergillus niger* and the yeast *Saccharomyces cerevisiae* are employed in these processes not only to unlock existing antioxidants but also to convert precursor molecules into new compounds with greater antioxidant potential [62,64].

#### 3.3.2. Heterologous Production via Genetic Engineering

An advanced strategy, known as heterologous production, utilizes genetically engineered microbes as “chassis organisms” or “microbial hosts” to synthesize compounds that are not native to them, such as plant-derived resveratrol [6,44]. Common hosts include *E. coli* and *Saccharomyces cerevisiae* [4]. This method can lead to dramatic increases in yield; for example, resveratrol can be produced at a concentration of 2.34 g/L in engineered bacteria, a stark contrast to the 7.95 mg/kg that can be extracted from its natural plant source [4].

#### 3.3.3. Downstream Processing: Recovering Intracellular Antioxidants

For many antioxidants like glutathione or Coenzyme Q10 that are produced and accumulate inside the microbial cells (intracellularly), the fermentation process is only the first stage. After the cultivation is complete, the target compound must be recovered and purified from the microbial biomass through a series of downstream processing steps. A typical recovery workflow includes

- Lysis (cell disruption)—The initial step involves breaking open the robust cell walls of the microorganisms to release their internal contents [38,39,40,41,42,43]. This can be achieved physically with methods like bead milling or by using thermal lysis, which involves heating the cells in water [38,39,40,41,42,43].
- Solvent extraction—Once the cells are broken, the desired antioxidant is extracted from the cellular debris using a suitable organic solvent.
- Purification—The final step involves purifying the crude extract through techniques like precipitation or crystallization to achieve a high-purity final product.

## 4. Antioxidants from Natural Sources

Obtaining antioxidants from natural sources is a foundational step for their application in the food, health, and beauty industries [38]. The field has seen a significant progression, moving from long-established, solvent-heavy extraction processes to more advanced, environmentally conscious “green” methods [36,65,66]. A key contemporary focus is the innovative use of agricultural and food industry by-products, which are increasingly recognized as rich repositories of these bioactive molecules [11,31,67]. This chapter examines the primary natural origins of antioxidants, contrasts traditional and novel procurement techniques, and clarifies the essential function of hydrolysis in releasing these valuable substances.

### 4.1. Origins of Natural Antioxidants

Antioxidants are found throughout the natural world and can be sourced directly from biological materials [13].

- Conventional botanical sources—Antioxidants are commonly found in fruits, vegetables, herbs, and spices [5]. For example, polyphenols are a major class of antioxidants broadly sourced from plants, including common beverages [11]. Specific sources mentioned in studies include the leaves of *Seseli gummiferum*, various vegetable oils like rice bran and coconut oil for vitamin E, and red ginseng oil [12,16,30].
- Agri-food industry by-products—A major sustainable trend involves the reclamation of antioxidants from industrial food processing waste [11,31]. This approach minimizes environmental impact by upcycling materials that would otherwise be discarded [67,68]. Prominent examples include
  ∘ Horticultural waste—Discarded materials like peels, leaves, and stems from fruits and vegetables serve as viable sources [67].
  ∘ Residues from winemaking—Grape pomace, which includes the skins and seeds left after pressing, is a potent source of polyphenols [67].
  ∘ Olive oil production waste—The by-products from producing extra virgin olive oil, especially the olive pulp, leaves, and wastewater, contain significant amounts of active phenolic compounds [67,68].
  ∘ Brewing industry by-products—Spent grains from brewing, such as barley and wheat, are rich in retained antioxidant compounds [39].
  ∘ Vegetable oil deodorizer distillates—These by-products from the oil refining process are the primary commercial source for natural vitamin E [36,58].

### 4.2. Extraction Technologies

The techniques used to isolate antioxidants from their natural matrices are typically split into two main groups: conventional methods and advanced green technologies [36,65,66].

#### 4.2.1. Conventional Extraction Techniques

These long-standing methods are still in use but are known for their significant limitations.

- Common methods—This group includes Soxhlet extraction, maceration, and steam distillation, all of which rely on a solvent to draw out the desired compounds from a solid material [31,36,64]. Simple hot-water soaks are also a traditional method for extracting phytochemicals [9,32].
- Major drawbacks—The primary criticisms of these techniques are their inefficiency, marked by extended processing times, substantial use of organic solvents that may be toxic, and high energy consumption [36,64,69,70]. The heat involved can also cause thermolabile antioxidant compounds to degrade [64,70].

#### 4.2.2. Green and Advanced Extraction Technologies

These modern approaches are engineered to be more rapid, effective, and environmentally sound [63,70,71].

- Ultrasound-Assisted Extraction (UAE): This technology employs high-frequency sound waves to induce acoustic cavitation, a process where microscopic bubbles form and violently collapse [36,64,70]. This action perforates plant cell walls, improving solvent access and leading to faster, higher-yield extractions [65,66].
- Microwave-Assisted Extraction (MAE): This method uses microwave radiation to create rapid, targeted heating within the plant sample [65]. The resulting internal pressure causes cell structures to rupture, which forces the bioactive compounds out into the solvent [64,70].
- Supercritical Fluid Extraction (SFE): This technique utilizes a substance, typically carbon dioxide (CO_2_), that is heated and pressurized beyond its critical point to act as a solvent [36,64]. It is regarded as a green technology because CO_2_ is non-toxic and is easily evaporated from the final extract [65,66]. A key limitation is its effectiveness primarily for non-polar compounds; extracting polar molecules may necessitate the addition of a co-solvent like ethanol [64,70].
- Pressurized Liquid Extraction (PLE): Also known as accelerated solvent extraction, PLE operates by using common solvents at high temperatures and pressures [36,64]. These conditions enhance the solvent’s ability to dissolve compounds and move through the material, dramatically cutting down on extraction time and the amount of solvent needed [65].
- Pulsed Electric Field (PEF) Extraction: A non-thermal technology, PEF applies brief, powerful bursts of electricity to the material [65]. This creates pores in cell membranes through electroporation, allowing intracellular antioxidants to be released without the use of heat [65].

### 4.3. The Critical Role of Solvents

The selection of a solvent is a paramount factor in the extraction process. The choice depends on the target antioxidant’s chemical nature; polar solvents like water, methanol, or ethanol are effective for water-soluble compounds such as phenolics, while non-polar solvents like hexane are used for fat-soluble compounds like carotenoids. Reflecting the trend toward sustainability, greener solvents are being adopted, including Natural Deep Eutectic Solvents (NADESs), which are made from non-toxic, natural components [40]. In some cases, industrial platforms have been developed to use only water for extracting polyphenols from waste materials like those from olive and grape processing [67].

### 4.4. Hydrolysis as a Preparatory Step for Antioxidant Release

Hydrolysis is frequently employed as a preparatory treatment to dismantle complex plant structures and free antioxidants that would otherwise be inaccessible [62,64].

- Enzymatic hydrolysis—This approach uses specific enzymes, such as cellulases and pectinases, to selectively break down components of the plant cell wall [62,64]. It is valued for being a gentle and precise method that effectively liberates bound phenolic compounds without causing them to degrade [62].
- Chemical hydrolysis—While treatment with acids or alkalis can also release bound antioxidants, these methods are often considered less ideal due to the aggressive conditions, which can damage the target compounds [62,64].
- Hydrolysis for substrate preparation—Beyond direct extraction, hydrolysis is also used to process complex biomass like agricultural waste (lignocellulose) into simpler sugars [14,35]. These sugars can then serve as an inexpensive feedstock for microbial fermentation processes [14].

## 5. Chemical Synthesis of Antioxidants

Laboratory-based chemical production has traditionally served as a primary pillar for the mass manufacturing of antioxidants, especially vitamins [36,72]. Although it is a well-established industrial component, this approach faces growing competition from more sustainable biotechnological methods [14,36,37]. Chemical synthesis can carry environmental costs, such as the use of hazardous materials and the creation of unwanted by-products [6,18,45]. Furthermore, synthetic pathways can yield molecules with different three-dimensional structures than those found in nature, a factor that profoundly impacts their biological effectiveness [13,36].

### 5.1. Manufacturing of Key Antioxidant Vitamins

#### 5.1.1. Vitamin C via the Reichstein Process

The foundational industrial pathway for producing vitamin C is the Reichstein process, a semi-synthetic method first established in the 1930s [24,36]. This hybrid approach starts with D-glucose and integrates a series of chemical transformations with a single, indispensable microbial fermentation step [24,36]. In this biological stage, the bacterium Gluconobacter oxydans is employed to convert D-sorbitol into L-sorbose, which is then chemically processed through esterification and lactonization to create the final ascorbic acid molecule [24,36,37].

#### 5.1.2. Synthesis of Vitamin E (Tocopherols)

The industrial production of vitamin E is achieved by chemically condensing two precursor molecules: trimethylhydroquinone (TMHQ) and isophytol [36,58]. The raw materials for these precursors are often sourced from fossil fuels, and the synthesis pathway can be complex and involve unpredictable reaction conditions [58,59].

A crucial outcome of this synthetic method is the creation of all-racemic (all-rac)-α-tocopherol, which is a mixture containing equal parts of all eight possible stereoisomers [13,36]. This is fundamentally different from naturally sourced vitamin E, which is composed solely of the single, more biologically potent RRR-α-tocopherol stereoisomer [36,59]. To ensure stability for commercial use, the final synthetic tocopherol is typically converted into an ester derivative, such as all-rac-α-tocopheryl acetate [13,36,59].

#### 5.1.3. Nicotinamide (Vitamin B3)

A primary industrial pathway for nicotinamide production is chemical synthesis [72]. This route usually starts with the ammoxidation of 3-methylpyridine to form the essential intermediate, 3-cyanopyridine [61,72]. Subsequently, this intermediate undergoes chemical hydrolysis to yield nicotinamide, a step that often relies on a strong alkaline catalyst and requires high-temperature and high-pressure conditions, which can lead to by-product formation and wastewater issues [61].

### 5.2. Widely Used Synthetic Phenolic Antioxidants

Several synthetic compounds with phenolic structures are extensively used in the food industry as preservatives to inhibit lipid degradation and rancidity [5]. They function by scavenging free radicals and terminating oxidative chain reactions [7]. The most prevalent examples are

- Butylated Hydroxyanisole (BHA) [5,7,34,73];
- Butylated Hydroxytoluene (BHT) [5,7,34,73];
- Tertiary Butylhydroquinone (TBHQ) [5,7,34,73];
- Propyl Gallate (PG) [7,34,73].

Despite their widespread use, there are documented health concerns related to the decomposition of these compounds and the potential for contamination from chemical precursors used during their synthesis [5,34].

### 5.3. Synthesis Pathways for Other Notable Antioxidants

#### 5.3.1. Glutathione and Coenzyme Q10

Although laboratory synthesis methods for glutathione and Coenzyme Q10 exist, they are generally not employed for large-scale commercial production [18,49,50,51]. The chemical synthesis of these molecules involves intricate, multi-step processes that are typically characterized by high costs, low overall yields, and a reliance on hazardous chemicals, making them less practical and economically viable than microbial fermentation routes [18,49,50,51].

#### 5.3.2. Organoselenium Compounds

For the specialized class of organoselenium antioxidants, chemical synthesis represents the main avenue for both research and industrial production [4]. Scientific efforts in this area are concentrated on designing multi-step synthesis pathways to create novel compounds that possess high stability and an optimal therapeutic profile [4,74].

#### 5.3.3. Idebenone

The synthesis of idebenone, an analogue of ubiquinone, serves as a clear example of a targeted chemical production route [11]. An efficient two-step method has been devised, achieving a high total yield of 81% [47]. The synthesis begins with a Friedel–Crafts Acylation reaction, which is followed by a combined Williamson Ether Synthesis and Oxidation step to form the final idebenone molecule [47].

### 5.4. Additional Concepts in the Synthesis of Antioxidants

#### 5.4.1. The “Natural-Identical” Category

A specific classification exists for molecules that are created through chemical synthesis but are structurally indistinguishable from their naturally occurring counterparts [73]. This group includes some of the most well-known antioxidants, such as ascorbic acid (vitamin C), β-carotene, and tocopherols (vitamin E) [73].

#### 5.4.2. Synthesis of Bioactive Polysaccharides

In addition to smaller molecules, chemical synthesis has been used to create synthetic polysaccharides [31]. Research has demonstrated that some synthetic oligosaccharides possess significant radical-scavenging capabilities and can elicit immune-stimulating responses comparable to those of natural glucans [32]. The chemical technique of carboxymethylation, which is based on the Williamson synthesis, is one method used for these modifications [32].

#### 5.4.3. Producing Nanoselenium

For antioxidant nanomaterials like nanoselenium, the principal production method is chemical reduction [41]. This involves reducing selenium salts with a reducing agent, such as ascorbic acid or cysteine, to form the elemental nanoparticles [75].

## 6. Advanced Biotechnological and Green Technologies in Antioxidant Production

The manufacturing of antioxidants is radically changing from traditional chemical synthesis toward innovative, sustainable, and highly efficient technologies [76,77]. This transition is fueled by a desire to mitigate the environmental footprint of conventional industrial methods and to meet a growing consumer demand for products derived from natural or “green” processes [11,78]. These modern platforms encompass a wide range of cutting-edge strategies, including cellular and genetic engineering, targeted enzymatic biocatalysis, novel physical processing techniques, and nanotechnology [52,63,67].

### 6.1. Genetic and Cellular Engineering Strategies

The manipulation of biological systems at the genetic level is a powerful cornerstone of modern biotechnology, enabling the development of customized systems for superior antioxidant output [36].

- Biofortification of transgenic plants: A primary application of genetic engineering is the biofortification of agricultural crops to enhance their nutritional content [36]. This is often accomplished by overexpressing pivotal genes within a plant’s natural antioxidant biosynthesis pathway [13]. For instance, by amplifying the expression of genes like VTE4 or HPPD in crops such as soybeans, researchers have successfully altered the plant’s metabolism to produce higher quantities of the most biologically active form of vitamin E, α-tocopherol [13,36].
- Development of engineered microorganisms: Using microbes as “cell factories” provides a highly controllable and sustainable manufacturing platform [72]. The advanced strategy of heterologous production involves inserting the genetic blueprint for a specific antioxidant into a host microbe, such as the bacterium E. coli or the yeast Saccharomyces cerevisiae [6,45]. This has led to major advancements, including
  ∘ The successful high-yield production of the polyphenol resveratrol in engineered microbes [6].
  ∘ The design of a novel one-step fermentation pathway for vitamin C by introducing a combination of plant and mammalian genes into yeast, which allows it to synthesize the vitamin directly from glucose [36].
- Induction of hairy root cultures: A specialized biotechnological tool involves using the bacterium Agrobacterium rhizogenes to genetically transform plant tissue, which induces the growth of hairy root cultures [6]. These cultures are highly prized as they are genetically stable and can produce secondary metabolites, including antioxidants, with high efficiency in a contained bioreactor environment [6].
- Strain enhancement through physical mutagenesis: In addition to precise gene editing, physical methods can be used to induce mutations and select for improved microbial strains [14]. For example, exposing the bacterium Gluconobacter oxydans to gamma radiation has been shown to generate mutant strains capable of producing nearly twice as much ascorbic acid as the original parent strain [12].

### 6.2. Biocatalysis and Enzymatic Processes

Enzymes, as nature’s biocatalysts, are foundational to both the biological synthesis and the biotechnological handling of antioxidant compounds [78]. These highly specific proteins drive complex chemical reactions under gentle conditions [78]. Their role in this field is twofold: they serve as the architects for the de novo creation of antioxidants within cellular systems, and they act as precision instruments for the extraction and modification of these molecules from natural sources [62,78].

#### 6.2.1. The Body’s Natural Enzymatic Defenses

To understand the importance of enzymatic antioxidants, it is useful to consider the body’s innate defense network, which is regulated by DNA [1,2]. This protective system is structured in tiers:

- Primary defense enzymes—This group represents the most potent defense against reactive oxygen species [2,5]. It is composed of superoxide dismutase (SOD), a powerful metalloenzyme that is a first line of defense; catalase (CAT), which requires an iron or manganese cofactor to neutralize hydrogen peroxide; and glutathione peroxidase (GPx), a selenium-dependent enzyme that degrades both hydrogen peroxide and lipid peroxides [2,7].
- Secondary defense enzymes—These enzymes, including glutathione reductase (GRd), function to support the primary defense system [5].

Industrially, some of these enzymes, like microbial catalases, can be produced using genetically modified strains of fungi [7].

#### 6.2.2. Enzymatic Pathways for Antioxidant Biosynthesis

The formation of antioxidants within living cells is entirely governed by enzymatic pathways, which are the primary targets for optimization in biotechnological manufacturing [44,49,50,51].

- Glutathione synthesis—This tripeptide is assembled from its three amino acid constituents via a two-stage, energy-dependent enzymatic construction [49,50,51]. The first reaction, catalyzed by γ-glutamylcysteine synthetase (GCS), is the rate-determining step in the pathway [49,50,51]. The final step, the addition of glycine, is catalyzed by glutathione synthetase (GS) [49,50,51].
- Coenzyme Q10 (CoQ10) synthesis—The microbial synthesis of CoQ10 is an enzyme-driven process that begins with two main precursor molecules [18,56]. Key catalysts include Decaprenyl Diphosphate Synthase (DPS), which builds the molecule’s isoprenoid side chain and is a rate-limiting enzyme, and 4-hydroxybenzoate decaprenyltransferase (UbiA), which performs the first committed step of attaching the side chain to the head group [18,56]. The final modifications to the molecule are carried out by a series of enzymes belonging to the ubi gene cluster [18,56].
- Carotenoid synthesis—This pathway starts from the central precursor isopentenyl pyrophosphate (IPP) [31]. A sequence of enzymes then builds the final carotenoid structure, including Phytoene synthase (CrtB) to create the initial C40 backbone, Phytoene desaturase (CrtI) to form lycopene, and Lycopene cyclase (CrtY) to create β-carotene [44]. The final conversion to xanthophylls like astaxanthin is performed by enzymes such as β-carotene hydroxylase (CrtZ) and β-carotene ketolase (CrtW) [44,53]
- Tocopherol (vitamin E) synthesis—In plants, tocopherols are synthesized within the plastids through a well-defined enzymatic pathway [13,43]. The process involves enzymes like p-hydroxyphenylpyruvate dioxygenase (HPPD) to create the aromatic head group and homogentisate phytyltransferase (HPT/VTE2) to attach the phytyl side chain [13,41].

The crucial final step, which converts γ-tocopherol into the highly active α-tocopherol, is catalyzed by γ-tocopherol methyltransferase (γ-TMT/VTE4) [13,43].

#### 6.2.3. Enzymatic Biotransformation and Molecular Modification

Beyond de novo synthesis, enzymes are used as powerful biocatalysts to transform simple precursors into valuable antioxidants or to alter the properties of existing ones.

- Nicotinamide and nicotinic acid production—The biotechnological production of nicotinamide hinges on the microbial enzyme nitrile hydratase, which efficiently converts 3-cyanopyridine to nicotinamide in a single step [61]. A second enzyme, amidase, can then hydrolyze the nicotinamide to produce nicotinic acid [60]. In industrial settings, these enzymes are often immobilized on a solid support to enhance their stability and reusability, which lowers overall production costs [60,61].
- Lipophilization of antioxidants—Enzymes, especially lipases, are utilized in low-water environments to increase the fat solubility (lipophilicity) of certain antioxidants [78]. This process of lipophilization, which involves creating esters of compounds like phenolic acids, can improve an antioxidant’s solubility in lipids and may even boost its biological effectiveness [78].

#### 6.2.4. Enzyme-Assisted Extraction (EAE)

EAE represents a green technology that employs enzymes to improve the recovery of antioxidants from natural materials, such as the by-products of food processing [62,64].

- Mechanism of action—This technique uses a targeted mixture of enzymes, including cellulases, pectinases, and proteases, to catalyze the digestion of the structural components of plant cell walls [62,64]. By dismantling the plant matrix, these enzymes facilitate the release of otherwise inaccessible bioactive compounds like polyphenols [62,64].
- Key advantages—EAE is considered a “green” method because it functions under gentle pH and temperature conditions, thereby protecting heat-sensitive antioxidants from degradation [62,64]. The high specificity of the enzymes can also result in a cleaner extract with fewer impurities [62,64]. For even greater efficiency, EAE is sometimes combined with other green technologies, such as ultrasound [42].

### 6.3. Plant Cell Culture as a Biotechnological Platform for Antioxidant Production

Plant cell culture represents a sophisticated biotechnological method for the contained and sustainable manufacturing of high-value phytochemicals, including antioxidants [6,77]. This technology is based on the in vitro cultivation of plant cells, tissues, or organs on a sterile, nutrient-rich medium [43]. A key advantage of this platform is its capacity to provide a consistent and predictable supply of these compounds, effectively bypassing the geographical, seasonal, and climatic variables that constrain traditional agriculture [6,77,79]. These systems serve a dual purpose, functioning as both a direct source for the production of valuable antioxidants and as an essential research tool for investigating their complex biosynthetic pathways [13,43].

#### 6.3.1. Types of Plant Cell Culture Systems

Different in vitro cultivation techniques can be employed, each with specific advantages for research and production.

- Callus and cell suspension cultures—Plant cells can be grown as an undifferentiated, unorganized mass known as a callus on a solid growth medium [6,77]. Alternatively, they can be cultivated as freely moving single cells or small cell clusters within a liquid medium, a setup known as a cell suspension culture [6,77]. Cell suspensions are especially advantageous for scaling up production in large industrial bioreactors and have been successfully used to produce antioxidants like ginsenosides and shikonine [6].
- In vitro root cultures—Since roots are often a plant’s primary site for synthesizing phytochemicals, they can be cultivated independently in vitro [6]. Adventitious roots and genetically transformed hairy roots can be grown for the continuous and genetically stable production of metabolites [6]. Notably, hairy root cultures have been employed to generate high yields of antioxidants like rosmarinic acid [6].
- Micropropagation—This technique, also called in vitro propagation, is used to generate a large number of genetically identical plantlets from a small piece of tissue [6]. These cloned plantlets can then be harvested for the extraction of their compounds [6]. In some instances, these micropropagated plants have demonstrated higher concentrations of phenolics and greater antioxidant activity than their wild-grown counterparts [6].

#### 6.3.2. Applications in Antioxidant Production and Research

Plant cell cultures are a versatile platform with applications ranging from direct manufacturing to fundamental scientific discovery.

- Direct production of antioxidants—While not as established as microbial fermentation for every compound, plant cell culture is a viable pathway for producing certain antioxidants. For example, Coenzyme Q10 has been successfully produced in vitro using cell cultures derived from various plants, including tobacco, safflower, and groundnut [44].
- A tool for biosynthetic pathway research—These controlled systems are an invaluable tool for scientific investigation into how antioxidants are made [13,43]. They have been instrumental in understanding the complex biosynthesis of vitamins. For example, the pivotal enzyme HPPD in the vitamin E biosynthetic pathway was first discovered using carrot cell cultures [13,43]. Furthermore, soybean and safflower suspension cultures have been used in feeding experiments to identify the precursors and determine the rate-limiting steps in tocopherol synthesis [13,43].

### 6.4. Advanced Green Processing and Synthesis

This area encompasses a suite of technologies designed to be more efficient and ecologically responsible than conventional methods.

- Green extraction technologies—Several advanced physical methods are now prioritized for their ability to reduce or eliminate the need for toxic solvents and to lower energy consumption [63,65,66]. These include ultrasound-assisted extraction (UAE), microwave-assisted extraction (MAE), supercritical fluid extraction (SFE), and pulsed electric field (PEF) extraction [62,65,68,76].
- Sustainable feedstocks and waste valorization—A core principle of the green approach is the concept of a circular economy, where waste is redefined as a resource [67]. This includes the valorization of low-cost agro-industrial wastes, such as fruit peels and lignocellulosic biomass, which can be used as nutritious substrates for the microbial fermentation of antioxidants [14,38,39,40,42,43].
- Application of green solvents—A major focus of green chemistry is the replacement of volatile organic compounds with environmentally safe alternatives [65,66,76]. Besides well-known green solvents like water and ethanol, innovative “designer” solvents such as Natural Deep Eutectic Solvents (NADESs) are gaining attention for being biodegradable, non-toxic, and derived from natural components [62,80].
- Green synthesis of nanoparticles—In nanotechnology, green synthesis protocols use natural extracts from plants or microbes as a source of antioxidants [75]. These natural compounds serve as effective reducing and stabilizing agents for the formation of metallic nanoparticles, thus replacing the toxic chemicals used in traditional synthesis [5,30].

### 6.5. Nanotechnology-Based Antioxidant Systems

Nanotechnology offers a frontier for designing novel antioxidant agents with superior performance [73].

- Intrinsically active nano-antioxidants—This approach involves using nanomaterials that possess their own inherent antioxidant capabilities [5]. Nanoparticles composed of cerium oxide or yttrium oxide, for instance, can function as regenerative radical scavengers by mimicking the activity of the body’s natural antioxidant enzymes [5].
- Antioxidant nanocarriers—Nanotechnology can also be used to enhance the stability and delivery of conventional antioxidants [73]. This is achieved by either encapsulating the antioxidant molecules within a protective nanocarrier or by coating them onto the nanoparticle’s surface [73].

## 7. Key Microorganisms in Antioxidant Production

A vast array of bacteria, yeasts, fungi, and microalgae is foundational to the production of antioxidants. These microscopic “cell factories” are leveraged in numerous ways, serving as the workhorses in industrial fermentations, providing the platforms for sophisticated genetic engineering, and acting as key agents in the sustainable bioprocessing of food industry wastes. This chapter will survey the specific microorganisms that are integral to manufacturing key vitamins and other valuable antioxidants, while also examining their wider applications in modern biotechnology and green chemistry.

### 7.1. Microorganisms in the Synthesis of Vitamins

#### 7.1.1. Vitamin C Manufacturing

The industrial pipeline for vitamin C production is a complex biological process that relies on the specialized functions of several microorganisms.

- *Gluconobacter oxydans*—This bacterium is the cornerstone of the initial production phase. It is tasked with the highly effective biotransformation of D-sorbitol into L-sorbose, a critical reaction in both the historic Reichstein process and contemporary two-step fermentation methods [37].
- *Ketogulonicigenium vulgare*—In the second stage of the two-step fermentation, this microbe is the principal producer [37]. It houses the complete enzymatic toolkit required to convert L-sorbose into 2-keto-L-gulonic acid (2-KLG), the immediate precursor of vitamin C [37,46]. Its own complex nutritional demands, however, mean it must rely on other microbes to thrive [37,46].
- *Bacillus* species (*B. megaterium*, *B. thuringiensis*)—These bacteria serve as crucial “helper strains” in the fermentation co-culture [37]. While they do not synthesize 2-KLG, they excrete essential metabolites like amino acids, which foster the growth of K. vulgare and thereby amplify the overall product yield through a synergistic interaction [37,46].
- *Saccharomyces cerevisiae* (yeast)—This well-characterized yeast is the primary organism of choice for engineering a more advanced, single-step fermentation route [47]. Scientists are working to equip this yeast with a synthetic pathway composed of genes from both plants and animals, which would enable it to create vitamin C directly from glucose [47,48].

#### 7.1.2. Manufacturing of Vitamin E Precursors

Although vitamin E is a plant product, microorganisms are indispensable for the industrial-scale synthesis of its chemical precursors (Table 4).

- *Saccharomyces cerevisiae* (yeast)—In industry, this yeast is the preferred “chassis organism” for the high-yield production of β-farnesene, a key starting material for synthesizing the isophytol component of vitamin E [58].
- *Escherichia coli*—As a research tool, this bacteria is used as a host to express plant enzymes for studies of the vitamin E pathway and has also been engineered for farnesene production [45,58].
- *Synechocystis* sp.—This cyanobacteria served as a vital model organism that helped scientists identify several key genes within the tocopherol (vitamin E) biosynthetic pathway [81].

## 8. Conclusions

The comprehensive exploration of antioxidants in the preceding chapters highlights their vital role in health and industry, from their fundamental function in mitigating oxidative stress to their application in disease management and materials science. This review has detailed their classification, mechanisms, and the primary paradigms for their production: natural extraction, chemical synthesis, and biotechnology. By surveying the array of advanced engineering strategies being deployed, a clear picture of the future of the field emerges.

A central theme is the ongoing evolution of antioxidant production. The field is moving beyond its historical reliance on two distinct pillars: natural extraction from agricultural sources and large-scale chemical synthesis. Natural extraction is rapidly transforming, shifting from resource-heavy conventional methods to sophisticated green technologies that prioritize sustainability and the valorization of agri-food waste. In parallel, traditional chemical synthesis, while still important for certain vitamins, is increasingly assessed by its environmental footprint and the structural limitations of its products, such as the creation of racemic mixtures instead of the single, more potent isomers found in nature.

The future of antioxidant production is being powerfully redefined by biotechnology, which offers two distinct and complementary platforms for creating high-value compounds in controlled environments. The first is microbial fermentation, a robust and scalable approach that utilizes “cell factories” like the yeast *Saccharomyces cerevisiae* and various bacteria for the rapid synthesis of antioxidants like glutathione and vitamin C precursors. The second is plant cell culture, a sophisticated in vitro technology that provides a sustainable source of complex phytochemicals, independent of climate and geography. This platform serves a dual purpose, enabling the direct manufacturing of compounds like rosmarinic acid and also acting as an indispensable research tool for elucidating complex biosynthetic pathways.

This biotechnological frontier is propelled by a multidisciplinary application of advanced engineering. Core disciplines like metabolic and genetic engineering provide the molecular tools to rationally redesign the biosynthetic “software” within both microbial and plant cells. Strategies such as the overexpression of key enzymes and the use of modern genome-editing tools are overcoming natural yield limitations. These molecular-level interventions are supported by bioprocess engineering, which optimizes the physical “hardware” of bioreactors to maximize productivity.

The evolution of antioxidant science is aimed at solving the field’s most pressing challenges: improving bioavailability, ensuring the sustainability of production, and guaranteeing economic viability. The innovative strategies discussed—from nanoencapsulation for better delivery and enzyme-assisted extraction for gentle processing, to the use of elicitors to boost yields in plant cell cultures—represent the direct and powerful solutions to these challenges.

The multidisciplinary character of the industry for obtaining antioxidants can be seen in Table 5.

**Figure 4:**
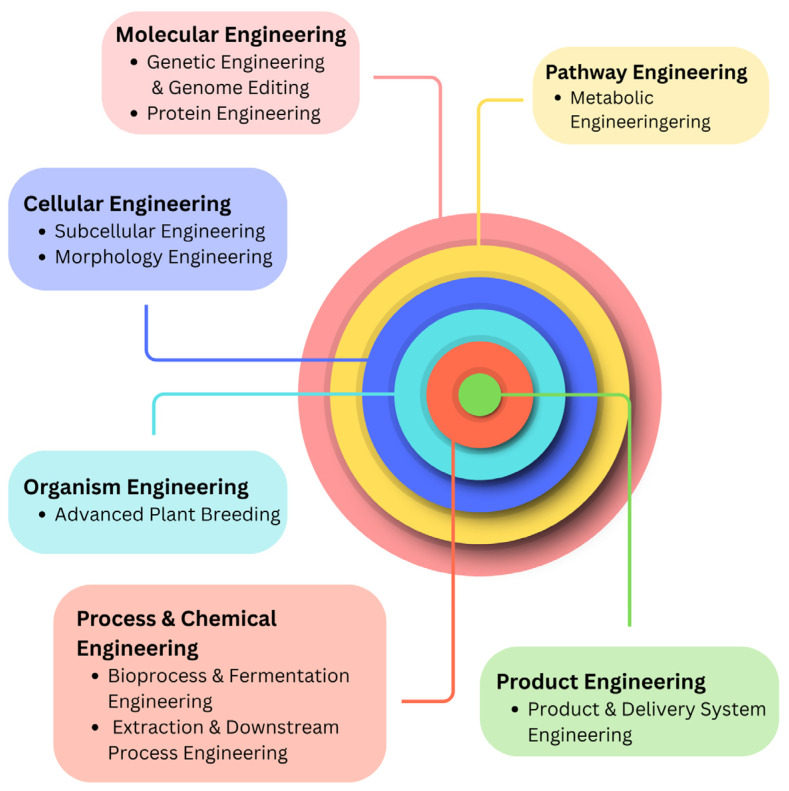
Multiscale engineering in producing antioxidants.

The progression from fundamental science to industrial application is represented in Figure 4.

In conclusion, the future of antioxidants will be defined by an integrated, systems-level approach. It will combine the precision of molecular biology in both microbial and plant systems, the sustainability of green chemistry, and the scalability of advanced process engineering. This synergy will not only enable the efficient production of known antioxidants but will also unlock the potential to create novel, bio-inspired molecules with enhanced stability and therapeutic efficacy, heralding the next generation of solutions for human health and industrial innovation.

## Figures and Tables

**Table 3:** Antioxidant compounds and their 2D chemical structures, according to PubChem https://pubchem.ncbi.nlm.nih.gov/ (accessed on 3 September 2025).

Antioxidant Compound	Chemical Structure
**Idebenone C_19_H_30_O_5_**	10-carbon side chain attached to a quinone ring 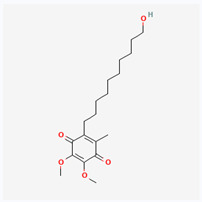
**Ubiquinone (Coenzyme Q10) C_59_H_90_O_4_**	A benzoquinone ring and a long, isoprenoid side chain containing 10 isoprene units, which makes it lipid-soluble. 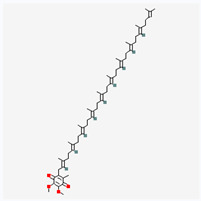
**Vitamin E (-)-alpha-Tocopherol C_29_H_50_O_2_**	A chromanol ring (a hydroxylated benzene ring fused to a dihydropyran ring) with a long phytyl side chain. The hydroxyl group on the ring is the active site. 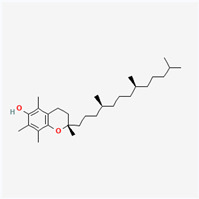
**Vitamin C (L-Ascorbic Acid) C_6_H_8_O_6_**	A six-carbon lactone with a furanose ring. It has several hydroxyl groups and a key enediol group (−C(OH)=C(OH)−), which is responsible for its antioxidant activity. 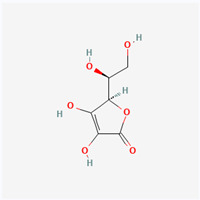
**Resveratrol C_14_H_12_O_3_**	A stilbenoid composed of two phenol rings linked by an ethylenic bridge. It exists in both cis and trans isomers. 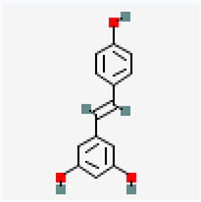
**Lycopene C_40_H_56_**	A linear tetraterpene with 11 conjugated double bonds, giving it its characteristic red color and high reactivity as a singlet oxygen quencher. 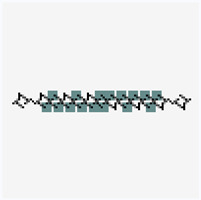
**Lutein C_40_H_56_O_2_**	A xanthophyll carotenoid, similar to lycopene but with two hydroxyl (−OH) groups and two cyclic end rings. 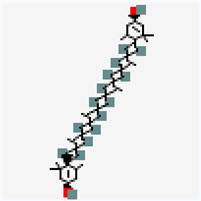
**Ferulic acid C_10_H_10_O_4_**	A hydroxycinnamic acid with a phenolic ring containing a hydroxyl and a methoxy group, and a conjugated acrylic acid side chain. 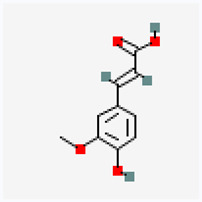
**Silymarin C_25_H_22_O_10_**	A complex mixture of flavonolignans; its main active component is silybin, which consists of a flavonoid unit and a coniferyl alcohol unit. 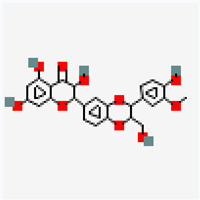
**Genisteine C_15_H_10_O_5_**	An isoflavone, with a chromen-4-one backbone and three hydroxyl (−OH) groups. 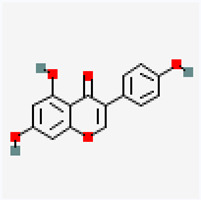
**Zeaxanthin C_40_H_56_O_2_**	A xanthophyll carotenoid and a stereoisomer of lutein, with the same chemical formula but a different arrangement of atoms at the ring ends. 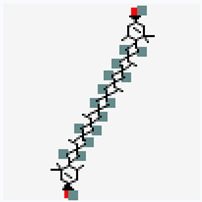
**Quercetin C_15_H_10_O_7_**	A flavonol characterized by two benzene rings and a heterocyclic pyran ring with five hydroxyl (−OH) groups. 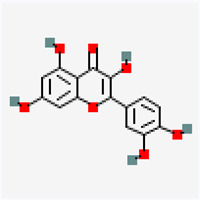
**Kaempferol C_15_H_10_O_6_**	A flavonol, similar to quercetin but with a different substitution pattern on its benzene rings. 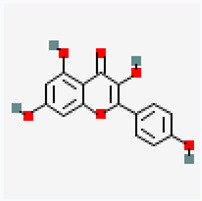
**Crocin C_44_H_64_O_24_**	A diester formed from the sugar gentiobiose and the dicarboxylic acid crocetin. It is a hydrophilic carotenoid. 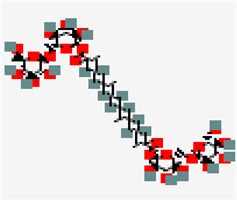
**Caffeic acid C_9_H_8_O_4_**	A hydroxycinnamic acid with a benzene ring containing two adjacent hydroxyl (−OH) groups and a carboxylic acid side chain. 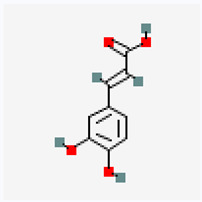
**Caffeine C_8_H_10_N_4_O_2_**	A methylxanthine alkaloid. Its structure is based on a purine ring with three methyl groups. 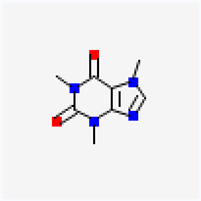
**Epigallocatechin gallate C_22_H_18_O_11_**	A catechin and an ester of gallic acid. It features a flavonoid skeleton with a galloyl group attached to a hydroxyl group. 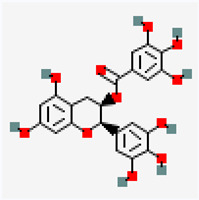
**Niacinamide (Vitamin B_3_) C_6_H_6_N_2_O**	A pyridine ring with a carboxamide group attached at the meta position. 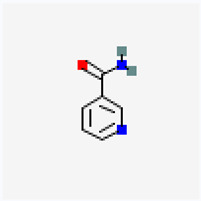

**Table 4:** Microorganisms used to produce other major antioxidants.

Application	Microorganism	Role and Key Details
**Vitamin C Production**	*Gluconobacter oxydans*	Performs the highly efficient bioconversion of D-sorbitol to L-sorbose, a critical first step in both the classic **Reichstein process** and modern **two-step fermentation** [37]
	*Ketogulonicigenium vulgare*	The primary producer in the second stage of the two-step fermentation; it possesses the full enzyme system to convert L-sorbose into the vitamin C precursor 2-keto-L-gulonic acid (2-KLG) [37,46]
	*Bacillus species* (*B. megaterium*, *B. thuringiensis*)	Act as indispensable **“helper strains”** that secrete essential nutrients to support the growth of *K. vulgare*, thereby enhancing the final product yield in a synergistic partnership [37,46]
	*Saccharomyces cerevisiae* (Yeast)	The main platform for engineering a more advanced, **one-step fermentation** process to produce vitamin C directly from glucose [47,48]
**Vitamin E Precursor Production**	*Saccharomyces cerevisiae* (Yeast)	The premier industrial host for the high-yield fermentation of **β-farnesene**, a key precursor for the chemical synthesis of isophytol [58]
	*Escherichia coli*	A versatile research host used to express plant enzymes to study the vitamin E pathway and for the engineered production of farnesene [58,81]
	*Synechocystis* sp.	A cyanobacterium that served as a vital model organism for identifying several key genes within the tocopherol (vitamin E) biosynthetic pathway [81]
**Glutathione Production**	*Saccharomyces cerevisiae* (Yeast)	The principal microbe used for the industrial-scale fermentation of glutathione, largely due to its GRAS (Generally Recognized as Safe) status [49]
	Other Yeasts (*Candida utilis*, *Pichia pastoris*)	Recognized as effective glutathione producers and serve as important alternatives to *S. cerevisiae* [49,50,51]
	*Escherichia coli*	A key host for the **heterologous production** of glutathione via advanced genetic and metabolic engineering techniques [49,50,51]
**Coenzyme Q10 Production**	*Agrobacterium tumefaciens and Rhodobacter sphaeroides*	The two most significant industrial bacterial producers of CoQ10, known for their high yields and are primary targets for metabolic engineering [53,54,55,56]
	*Escherichia coli*	A crucial host for the heterologous production of CoQ10, where it functions as a “cellular factory” that can be cultivated to very high cell densities [54,56,57].
**Carotenoid Production**	*Phaffia rhodozyma* (Yeast)	The most important commercial yeast source for the antioxidant **astaxanthin** [44,52]
	*Blakeslea trispora* (Fungus)	A major industrial producer of **β-carotene** and **lycopene** through fermentation [44]
	*Haematococcus pluvialis* (Microalga)	The primary commercial source of natural **astaxanthin**, which it accumulates under stress conditions [53]
	*Dunaliella salina* (Microalga)	The main commercial source of natural **β-carotene**, which it produces in response to high salinity and light stress [53]
	*Paracoccus carotinifaciens* (Bacterium)	A significant industrial producer of **astaxanthin** [44].
**Nicotinamide (Vitamin B3) Production**	*Rhodococcus species* (e.g., *R. rhodochrous*)	The primary source of the enzyme **nitrile hydratase**, which is essential for the efficient biotransformation of a chemical precursor into nicotinamide [60,72] (vitamin B3)
**Resveratrol Production**	*Escherichia coli and Saccharomyces cerevisiae*	The two most commonly used hosts for the **heterologous production** of this high-value polyphenol antioxidant [6,72]
**Fermentation of Food By-Products**	*Aspergillus niger and Rhizopus oligosporus*	These filamentous fungi are used in solid-state fermentation (SSF) to secrete enzymes that degrade the cell walls of food wastes (e.g., apple pomace), releasing bound phenolic antioxidants [62]
	*Lactobacillus plantarum and Bifidobacterium lactis*	These lactic acid bacteria have been shown to enhance the antioxidant properties of apple pomace through fermentation [62]
**General Bioprocessing Roles**	Actinomycetes, Cyanobacteria, and Lichens	These diverse microbial groups are known to naturally produce a wide range of unique secondary metabolites with potent antioxidant properties [5]
	Various Bacteria, Yeast, Fungi, and Algae	Extracts from these microorganisms are used as non-toxic reducing and stabilizing agents in the eco-friendly **“green synthesis”** of metallic nanoparticles [5]
	Yeast Extract	Used as a **biotic elicitor** in plant cell culture systems; it triggers a defense response in the plant cells, stimulating them to produce more antioxidant polyphenols [77]

**Table 5:** A survey of engineering disciplines in antioxidant production.

Engineering Discipline/Strategy	Description, Key Approaches, and Examples
**Metabolic Engineering** The deliberate and targeted redesign of an organism’s metabolic network to channel resources towards the synthesis of a specific antioxidant [6,13,18,44,49,50,51,56,82]	**Overexpressing key genes** **Blocking competing pathways** **Enhancing precursor supply**
**Genetic Engineering and Genome Editing** The direct manipulation of an organism’s genetic code to introduce new traits [13,36,41]	**Transgenic approaches** **Genome editing**
**Protein Engineering** This advanced discipline aims to improve the function of key enzymes by altering their physical structure [58,72]	Methods like **directed evolution**
**Subcellular and Morphology Engineering** These disciplines involve manipulating the physical structure and organization of the cell [45]	**Subcellular compartmentalization** **Morphology engineering**
**Advanced Plant Breeding** In addition to direct genetic modification, advanced breeding techniques that use molecular information are employed to improve antioxidant levels in crops [13,36,43]	**Quantitative Trait Locus (QTL) mapping** and **marker-assisted breeding**
**Bioprocess and Fermentation Engineering** This field is dedicated to optimizing the performance of large-scale microbial cultures in bioreactors [37,46,55,56]	Designing optimal high-density **fed-batch** or **continuous fermentation** strategies
**Extraction and Downstream Process Engineering** This discipline engineers the crucial steps of recovering and purifying the antioxidant after it has been produced [62,64,70]	**Process optimization**: Statistical modeling tools like **Response Surface Methodology (RSM)** **The biorefinery concept**
**Chemical and Materials Engineering** These fields contribute to the development of novel production methods [24,60]	**Chemical engineering** **Materials engineering**
**Product and Delivery System Engineering** This area focuses on engineering the final antioxidant product to enhance its stability and biological effectiveness [38,39,40,42,43,45]	**Nanoencapsulation** and **delivery system engineering** (involves designing **nanocarriers** that encapsulate the antioxidant) **Product sequestration and secretion**

## Data Availability

All data provided in this material can be found in the indicated references.
